# Development and validation of the short form domestic elder abuse assessment questionnaire (SF-DEAQ)

**DOI:** 10.1186/s12877-023-04388-x

**Published:** 2023-10-12

**Authors:** Amirreza Zobdeh, Razieh Bandari, Majideh Heravi-Karimooi, Maryam Mashayekh, Maryam Hazrati, Ali Montazeri

**Affiliations:** 1 Faculty of Pharmacy and Pharmaceutical Sciences, Monash University, Melbourne, VIC Australia; 2 Social Determinants of Health Research Center, Semnan University of Medical Sciences, Semnan, Iran; 3 Elderly Care Research Center, Faculty of Nursing & Midwifery, Shahed University, Tehran, Iran; 4 Amir-Almomenin Hospital, Busher University of Medical Sciences, Genaveh, Iran; 5 Iranian Research Center on Aging, University of Social and Rehabilitation Sciences, Tehran, Iran; 6 Shiraz Transplant Center, Abu-Al Sina Hospital, Shiraz University of Medical Sciences, Shiraz, Iran; 7 Population Health Research Group, Health Metric Center, Iranian Institute for Health Sciences Research, ACECR, Tehran, Iran; 8 Faculty of Humanity Sciences , University of Sciences & Culture, Tehran, Iran

**Keywords:** Domestic elder abuse, Older adults, Reliability, Validity, Psychometric, Development

## Abstract

**Background:**

The present study aimed to design and determine the psychometric properties of a short-form questionnaire to investigate the domestic elder abuse.

**Methods:**

This study consisted of two phases: in phase 1 we employed a modified Delphi approach with 18 participants. Consequently, content and face validity, and item analysis were applied. In Phase 2 we evaluated structural validity and convergent validity. Reliability was assessed by looking at internal consistency, stability, and absolute reliability.

**Results:**

The findings led to the development of a 27-items short form of domestic elder abuse in four domains that jointly accounted for 74.14% of the variance observed. The short form showed high internal consistency (Cronbach’s alpha = 0.93) and significantly correlated (r = 0.91; p < 0.001 for both scales) with the comprehensive (49-item) domestic elder abuse.

**Conclusion:**

The short form of domestic elder abuse was found to be reliable and valid as the longer version. The short form of domestic elder abuse could lessen the burden on respondents.

## Background

Aging is becoming a global phenomenon in both developed and developing countries. The elderly population has increased rapidly during the past decades due to two critical factors: decrease in mortality and fertility rates and overall improvements in people’s quality of life [1, 2]. The World Health Organization (WHO) has predicted that the world’s elderly population will surpass 12–22% by 2050 [3, 4]. In 2050, 80% of the elderly will live in low- and middle-income countries, unprepared to face aging and its social and economic effects [5, 6].

The consequences of an increasing number of older adults in the family include adverse effects on families’ economic conditions, mental and emotional well-being, extra responsibility, decreasing tolerance, individual fatigue and social isolation of family members. These consequences can be followed by the emergence of anti-social behaviors and violence [7]. Under such circumstances, families are often unprepared to care for the older adults. Combined with other social factors such as urbanization, modernity, changing traditional values, and the contrast between the value systems of the new and old generations, families sometimes do not play their proper role for the older adults, who may be exposed to domestic elder abuse instead [8, 9].

The World Health Organization (WHO) and the International Network for the Prevention of Elder Abuse define elder abuse as: ‘Elder abuse is a single or repeated act, or lack of appropriate action, occurring within any relationship where there is an expectation of trust, which causes harm or distress to an older person’ [10]. The WHO reported that in 2017, around 1 out of 6 older people experienced some form of abuse [11], and the results of a meta-analysis in 2017 indicated that 15.7% of people aged 60 years and over underwent some form of abuse [12]. Elder abuse can be categorized according to the type of abuse: psychological, physical, sexual, and financial abuse and neglect [13].

Abuse often occurs in family settings at home [14, 15], and the first perpetrators of elder abuse are the family members [14]. Elder abuse is a multidimensional issue [15] where most studies have reported that being female, physical disability, functional impairment, dependence on others, poor physical or mental health, low income or poverty, and lack of social support were major risk factors associated with elder abuse [16–19].

Abuse exerts adverse effects on mental and physical capacity, and social elderly people [20] and is associated with negative outcomes, including mental distress, morbidity, and death [21, 22]. Furthermore, the issue is very costly regarding the possibility of transfer to a nursing home and hospitalization [23]. Thus, identifying domestic elder abuse before any damaging consequences seems essential.

Previously we have developed and validated a comprehensive domestic elder abuse assessment questionnaire. This questionnaire included (49-item): emotional neglect (2 items), care neglect (11 items), financial neglect (6 items), dependence (10 items), psychological abuse (8 items), ostracism (4 items), physical abuse (6 items), and financial abuse(4items). The response categories were ‘yes,’ ‘no,’ and ‘not applicable.’ The score ranged from 0 to 100, with higher scores indicating higher levels of domestic elder abuse [9]. However, since the questionnaire was long and could take time for completion, we thought to develop a short version of the questionnaire. Therefore, the present study was designed to develop a short version of the Domestic Elder Abuse Assessment Questionnaire (SF-DEAQ) and evaluate its validity and reliability.

## Methods

We performed a mixed-methods study (using quantitative and qualitative research approaches).

### Qualitative phase

We applied the modified Delphi method for item reduction. As such experts with the necessary expertise, experience, and motivation were sought. Delphi panelists were recruited by email using a convenience snowball sampling approach. Members of the panel were selected from experts in gerontology (n = 2), nursing (n = 2), psychiatry (n = 2), legal medicine (n = 2), sociology (n = 2), psychology (n = 2), law (n = 2), religious and Islamic sciences (n = 2), and social work (n = 2). After the initial selection, the list of panelists was shared with the research team to obtain additional recommendations for panelists, but no specific panelists were suggested. We used a consensus process with 18 experts in a five-round modified Delphi process to identify the most vigorous domestic elder abuse possibilities. We used web-based surveys (via the Google Docs survey platform for rounds 1–3 and 5) and an in-person meeting (round 4). Panelists unable to attend the in-person meeting could participate in real-time via webinar (using the Google Meet platform) or in advance via a web-based survey. Synchronous voting for in-person and webinar attendees was conducted using mobile devices. Each round (except the final round) was preceded by a presentation (recorded webinar in rounds 1–3 and in person for round 4) summarizing the results from the previous round and preparing panelists for the upcoming round. In addition, after rounds 1 to 3, panelists were provided with individual reports, which included each panelist’s response, the aggregate responses, and comments from the other panelists. During the Delphi process, panelists evaluated dimensions and definitions of domestic elder abuse to reach a consensus (> 80% agreement). In rounds 1 through 3, panelists were presented with each principle and definition (starting in round 2) and asked to keep, modify, or remove an item. If a modification or removal was selected, panelists were asked a follow-up open-ended question on their choice. In rounds 4 and 5, panelists were asked whether they agreed or disagreed with each domestic elder abuse definition. We considered 2 metrics from the Delphi panel process when creating the short form version of the domestic elder abuse: we asked panelists to rank the domestic elder abuse, with 7 being the most important (should to be included) and 1 being the least important (could be omitted). We also asked panelists to select the 3 domestic elder abuse with the most importance and the 3 domestic elder abuse with the least importance. Accordingly, three items were excluded giving a 46-items questionnaire. Then, it was subjected to content and face validity as follows:

*Content Validity*: Using the qualitative content validity method, fifteen experts in the fields of gerontology, family nursing, psychiatry, legal medicine, sociology, psychology, law, religious and Islamic sciences, social work, and questionnaire development were invited to review and give comments on the writing and presentation style of the questionnaire [24]. The edited questionnaire was sent back to the expert panel for final approval. For quantitative content validity, the same expert panel was requested to fill out specific forms to calculate the content validity ratio (CVR), the content validity Index (Item Content Validity Index: ICVI > 0.78 & Scale Content Validity Index/ Average: SCVI/Ave > 0.9). According to the Lawshe table, a CVR score above 0.42 indicates satisfactory content validity for the questionnaire [25]. The CVI determines items’ relevance, simplicity, and clarity using a 4-point Likert scale, and a CVI score above 0.79 is considered appropriate. A score greater than 0.90 for S-CVI/Ave at the questionnaire level is considered appropriate [26, 27]. At this stage in all 7 items were excluded.

*Face validity*: To examine the qualitative face validity of the domestic elder abuse questionnaire, the principal investigator (RB) conducted individual interviews with ten older people to identify their perspectives on the understandability and simplicity of the questionnaire’s items, and their suggestions were included. For quantitative face validity, a 5-point Likert scale was used to assess the questionnaire’s items from ‘strongly important’ (score 5) to ‘not at all important (score 1). The impact scores for each item were calculated, and values over 1.5 were considered appropriate [26, 27]. At this stage extra 8 items remove and the provisional version of the questionnaire with 31 items was provided.

*Initial item analysis*: Finally, we performed item analysis. It is the preliminary evaluation of the questionnaire in the target population before its widespread use for data collection. Item analysis is usually performed before evaluating the validity of the questionnaire’s structure. Fifty older people were selected using the convenience sampling method and were requested to complete the domestic elder abuse questionnaire. Each item in the questionnaire was evaluated for mean, standard deviation, correlation with other items, and internal consistency of the whole questionnaire. The loop method was used for item analysis by evaluating the reliability coefficient of the entire questionnaire. If reliability increased with the omission of each item, the item had an influential role in coordination with other items, so it was entered into different stages of psychometric evaluation. The basis for deleting the item is that the correlation of the item with the total item score should be < 0.3. Additionally, if Cronbach’s alpha was increased with the removal of an item, it showed that the item should be deleted. At this stage, Cronbach’s alpha was estimated to be 0.934; no items were deleted. Thus the provisional version of the questionnaire with 31 items was subjected to psychometric evaluation.

### Quantitative phase

A cross-sectional study was conducted to examine the psychometric properties of the short version questionnaire described as follows:

*Construct validity*: To assess construct validity, the following procedures were applied.

1. Structural validity: Factor analysis is one of the best methods to assess structural validity. Two hundred eighty five older adults who met the inclusion criteria (being aged 60 and older, having no hearing and vision deficit as per self-reports, no cognitive decline, obtaining a score of seven or higher in the Iranian version of the Abbreviated Mental Test by Foroughan et al. (28) [28], and willingness to participate in the study) were selected through convenient sampling. Exploratory factor analysis (EFA) will not extract factors (latent variables). A Kaiser-Meyer-Olkin (KMO) test was performed to see the adequacy of the samples. The Bartlett Test of sphericalness was utilized in the sample to confirm that the matrix underlying the correlational analysis is not zero. Values above 0.7 in the KMO test and p-values less than 0.05 in Bartlett’s test were thought-about because of the quality criterion for correlational analysis. This study used the maximum likelihood method with Promax rotation for data exploratory factor analysis.

2: Convergent validity: To compare the short form of the domestic elder abuse questionnaire to the comprehensive version, we calculated the Pearson correlation coefficient.

#### Relative reliability

Internal consistency, stability, and absolute reliability were assessed to determine the reliability of the measure. To measure the relative reliability, we assessed Cronbach’s alpha and the intraclass correlation coefficient (ICC). Internal consistency refers to the correlation between the items in a tool. An alpha value of 0.7 or higher was considered for measuring the internal consistency. In addition to estimating the intraclass correlation coefficient (ICC), 30 participants completed the questionnaire twice with an interval of two weeks. A correlation coefficient of 0.8 or higher was considered satisfactory [29, 30].

*Absolut reliability*: The minimal detectable change (MDC) and standard error of measurement (SEM) were calculated to measure absolute reliability. The following equation was used to calculate the standard error of measurement: SEM = SD√1–ICC. To calculate the MDC, we used the following equation: MDC = SEM×Z×√2. The MDC can be calculated as a percentage to determine further the relative changes after treatment or between repeated measurements over time to show the relative value of the random measurement error. MDC %= (MDC ÷ mean) ×100, where the mean is the mean score of all repeated measurements [31]. MDC percent is considered acceptable if it is less than 30%, while the excellent MDC percent value is considered lower than 10% [31–33].

*Interpretability*: The benchmarks for the interpretability, according to the COSMIN (Consensus-based standards for the selection of health measurement instruments) checklist, are calculating the minimal important change (MIC), detecting ceiling and floor effects, describing the distribution of total scores in samples, and detect the percentage of missing items and the appropriateness of the sample size [34]. As a result, interpretability was investigated utilizing several methodologies, as follows.

- Minimal important change-MIC: For calculating MIC, the standard deviation of the change between the test-retest scores is multiplied by the mean effect size, which is 0.50 [35]. The MIC should be larger than the. MDC.
- One of the criteria for interpretability is the desired ceiling and floor effect. The total score of the questionnaire was set between numbers from one to 100. Accordingly, it was determined how many percent of the participants scored either zero or 100. Also, the ceiling and floor effect was calculated separately for each domain. This index should be less than 20% [36], although there is no agreement among researchers, and some believe it should be more than 15% [37].
- The frequency of non-responded (missing) elements is another technique used to confirm the interpretability of the dimensions. That is ideal if the value falls between 15% and 20% [38]. Replacing the missing data with the mean score is one technique to manage the missing data [39]. This alternative strategy was applied. However, efforts were made to reduce missing items by requesting participants to share their knowledge by answering as many questions as possible.

*Feasibility*: The definition of feasibility or ease of use is an instrument’s simplicity of retrieval and usefulness in assessing the relevant construct [31]. In this investigation, the frequency of the responses and the frequency of the items that were not answered was identified for each question, and an accurate factor analysis was carried out to avoid the need for a comprehensive inventory.

*Scoring*: Each item is rated on a 3-point Likert scale (‘yes,‘ ‘no,‘ and ‘not applicable). Option impractical indicates that the desired phrase is incompatible with older adult living conditions. The scores range from 0 to 100, with higher scores indicating higher domestic abuse levels. To calculate scores, the following formula was used: (Number of yes answers*/ (*total items-NA items)) *100.

## Results

### Participants

In all, 284 older adults took part in the study. Of these, 149 (52.4%) were female, 73.2.0% (n = 208) were married, and 49.6.0% were housewife. Most participants reported that they are living with wife/ spouse (65.1%) and indicated themselves as having intermediate economic status (42.1%). The characteristics of participants are shown in Table 1.

**Table 1:** The characteristics of study participants (n = 284)

		Number (%)
Gender		
	Man	135(47.6)
	Female	149(52.4)
Age group (years)		
	60–69	154(54.2)
	70–79	92(32.4)
	> 80	38(13.4)
Mean (SD)	69.46 (SD = 8.47)	
Educational		
	Illiterate	143(50.4)
	Primary	56 (19.7)
	Secondary	47(16.5)
	Diploma	25(8.8)
	Higher	13(4.6)
Marital status		
	Married	208(73.2)
	Single	1(0.4)
	Widowed	70(24.6)
	Divorced	5(1.8)
Employment status		
	Housewife	141(49.6)
	Employed	2(0.7)
	Retired	99(34.9)
	Unemployed	42(14.8)
Number of children		
	1–3	80(28.2)
	4–7	149(52.5)
	8–10	48(16.9)
	> 10	7(2.5)
Living condition		
	Alone	34(12)
	with spouse/wife	185(65.1)
	With children	5(1.8)
	With his spouse and children	60(21.1)
Economic status		
	Poor	73(25.3)
	Intermediate	134(42.1
	Good	77(32.7)
Housing		
	The owner	245(86.3)
	Tenant	23(8.1)
	Children’s home	16(5.6)
Health status		
	Very Poor/poor	18(6.4)
	Fair	115(33.2)
	Good/very good	151(60.5)
Insurance		
	Yes	271(95.4)
	No	13(4.6)

### Factor analysis

Exploratory factor analysis was used to assess the structural validity. Promax rotation and the maximum likelihood method were used to create the clusters. The appropriate sample size was estimated using the Kaser-Meyer-Olkin (KMO) value, and the item correlation matrix was examined using the Bartlett test for sphericity. KMO was found to be 0.91. Also significant at the 0.0001 level was Bartlett’s test for sphericity, which yielded a value of 8993.517. The findings pointed to a four-factor explanation that, taken together, accounted for 74.14% of the overall variance seen. The questionnaire now only has 27 items because four of them were eliminated at this point due to low loading (lower than 0.3). Table 2 presents the result.

**Table 2:** Exploratory factor analysis of the Short-form Persian Version Domestic Elder Abuse Assessment Questionnaire (n = 284)

Items	F 1	F 2	F3	F4
38.Has a family member tried to choke you?	**0.984**	0.138	0.020	0.181
37.Has a family member thrown an object at you?	**0.954**	0.138	0.061	− 0.072
36.Have you ever been hit, slapped, punched, pushed, pinched, pulled by the hair, or hit with objects such as a belt, a whip, or a stick?	**0.931**	0.128	-0.062	0.232
35.Has anyone mocked you or used offensive body language?	**0.907**	0.254	0.163	0.134
39.Has a family member tried to sedate you using medications or drugs?	**0.821**	− 0.004	0.144	0.029
33.Have you ever been called names or been addressed with an offensive tone or language?	**0.688**	0.117	0.033	0.205
34.Has a family member ever yelled at you?	**0.586**	-0.037	-0.065	0.181
32.Have you been blamed for no reason?	**0.523**	0.012	0.048	− 0.072
8.Have you ever needed help using the toilet, but your family did not help you?	0.088	**0.943**	-0.148	0.232
11.Has any family members failed to accommodate your needed diet despite being able to afford it?	0.137	**0.916**	-0.123	0.134
6.Have you ever needed help to get your medicines from the pharmacy or needed support to take your medications, but your family did not help you?	0.108	**0.893**	-0.130	0.029
5.Have you ever needed help to attend a medical appointment, but your family did not help you?	0.020	**0.862**	0.156	0.048
10.Has you ever failed to receive food or fluids in time?	0.138	**0.798**	0.202	0.033
4.Have you ever needed help to eat or drink, but your family did not help you?	0.138	**0.791**	0.072	-0.171
3.Have you ever needed help to move within the house, but your family did not help you?	0.128	**0.783**	-0.172	0.115
12.Have you ever needed help running errands, e.g. shopping or paying bills, but your family did not help you?	0.254	**0.695**	0.126	0.198
13.Have you ever needed help for house chores, e.g., cleaning or maintenance, but your family did not help you?	− 0.004	**0.678**	0.033	0.137
7.Have you ever needed help for personal hygiene or showers, but your family did not help you?	− 0.037	**0.576**	0.243	0.012
9.Have you ever needed any care products such as glasses, dentures, hearing aids, walking sticks, 4-wheel walkers, or a wheelchair, but your family has failed to purchase them for you?	0.088	**0.453**	0.238	0.182
17.Have you ever needed any comfort accessories, such as a comfortable bed, but your family has failed to purchase them for you?	0.137	0.005	**0.942**	0.153
16.Have you ever needed financial help to get a gift for someone, but your family did not help you?	0.108	0.241	**0.900**	0.049
14.Have you ever needed financial help for necessities, but your family did not help you?	0.020	0.028	**0.849**	0.006
15.Has any of your family harped on their financial help?	0.109	0.261	**0.594**	0.019
48.Have you ever been taken to the hospital and haven’t been visited in the hospital?	0.148	0.029	0.115	**0.890**
47.Have you ever been forced out of your own house?	0.171	0.048	0.000	**0.869**
49.Has your family taken you to the nursing home and failed to visit you in a while?	0.202	0.020	-0.106	**0.838**
46.Has a family member forced you out of their house?	0.240	0.065	-0.018	**0.776**
*Eigenvalue*	9.417	6.892	2.297	1.412
*% Variance*	34.879	25.527	8.507	5.231

F1: Physical and psychological abuse, F2: Care neglect, F3: Financial neglect, F4: Ostracism

### Convergent validity

Table 3 shows correlations among the short form and comprehensive measures for convergent validity. The two versions of domestic elder abuse showed significant correlations and support in terms of convergent validity (r:0.637–0.713) P < 0.01.

**Table 3:** The correlation between the short form of the domestic elder abuse questionnaire to the comprehensive version(n = 150)

	Comprehensive version	Care neglect	Financial neglect	Physical and psychological abuse	Ostracism	Total
Care neglect	0.713^**^	1				
Financial neglect	0.637^**^	0.509^**^	1			
Physical and psychological abuse	0.657^**^	-0.041	0.023	1		
Ostracism	0.695^**^	0.153	0.266^**^	0.375*8	1	
Total	0.911^**^	0.863^**^	0.707^**^	0.365^**^	0.432^*8^	1

^**^Correlation is significant at the 0.01 level

### Relative reliability

The Cronbach alpha values obtained for each factor were desirable, as shown in Table 4. In addition, the whole scale and the subscales had ICCs of 0.98, ranging from 0.92 to 0.98.

**Table 4:** Cronbach’s Alpha coefficient by dimensions and the whole questionnaire

Factor	No. of items	Cronbach’s alpha	ICC(N = 30)
Care neglect	11	0.94	0.98
Financial neglect	4	0.92	0.92
Physical and psychological abuse	8	0.94	0.97
Ostracism	4	0.98	0.93
Total	27	0.93	0.98

### Absolut reliability

The standard measure means error calculation was used to evaluate absolute reliability. Additionally, the estimated minimal detectable change (MDC) was given. Table 5 shows the result.

**Table 5:** The Absolute Reliability of Domestic Elder Abuse Assessment Questionnaire

Factor	mean	SD ^a^	ICC^b^	CI^c^= 95%	P-value	SEM^d^	MDC^e^	MDC%	Result
Care neglect	18.42	4.21	0.98	0.95–0.99	P < 0.001	0.595	1.64	9.17	Excellent
Financial neglect	16.40	5.74	0.93	0.82–0.96	P < 0.001	1.49	4.12	25.12	Optimal
Physical and psychological abuse	18.12	2.23	0.97	0.95-0.99	P < 0.001	0.386	1.06	5.84	Excellent
Ostracism	8.18	2.81	0.93	0.83–0.97	P < 0.001	0.730	2.0.2	24.69	Optimal
Total	22.19	7.07	0.98	0.96-0.99	P < 0.001	0.999	2.76	12.43	Optimal

a: Standard Deviation; b: Interclass Correlation Coefficient; c: Confidence Interval; d: Standard Error of Measurement; e: Minimal Detectable Changes

### Interpretability

According to the formula below, the average effect size of 0.5 should be multiplied by the standard deviation of the variations between the test-retest in order to get the MIC. MIC = 0.5 × SD of the Δ Score. the MIC must be larger than the MDC [31]. Considering that the standard deviation of the test-retest score was 7.07, the multiplication value was 3.53, which was higher than the MDC value. For each factor, the ceiling and floor effects were separately determined. For the whole questionnaire, the ceiling and floor effects were zero; for the subscales, they were below 20%, which is acceptable.

### Feasibility

The questionnaire took between 15 and 20 min to complete, on average.

## Discussion

The research led to the development and evaluation of the short-form domestic elder abuse assessment questionnaire. The process of psychometric properties’ evaluation of this questionnaire complied with the COSMIN checklist as a consensus-based standard on the properties of instruments for the measurement of health status [40].

It is important to note that the questionnaire is relatively simple; nurses or other healthcare professionals can complete it in around 15 min in various settings, including hospitals, clinics, rehabilitation facilities, and nursing homes. The validity of face and content confirmed the simplicity and clarity of statements. Although during the validation process of the questionnaire, due to the illiteracy or low literacy of the majority of the older adults, it was read to the subjects to standardize how they complete each item. However, due to its simplicity, clarity, and expression, it is quite possible and simple for literate people to complete the questionnaire. After the validity and reliability phases were completed., domestic elder abuse consisted of 27 items and four dimensions. The dimensions included physical abuse, psychological abuse, care neglect, financial neglect, and ostracism.

Physical abuse is a profound violation of human rights that leaves its victims with visible wounds and unseen scars that may last a lifetime. Acknowledging the lasting impact of physical abuse is vital to develop effective strategies for prevention and support. By raising awareness and offering empathy and compassion, we can contribute to breaking the cycle of violence and helping survivors reclaim their lives, fostering a society free from the devastating effects of physical abuse [41]. Often concealed within interpersonal dynamics, psychological abuse has significant implications for individuals’ mental well-being and dignity, especially older adults. The cultural context plays a vital role in shaping how such abuse is perceived and recognized, with traditional Eastern societies and Western cultures exhibiting divergent viewpoints. Acknowledging these cultural nuances and promoting respectful communication and empathy are essential steps toward fostering healthier relationships and dismantling the hidden menace of psychological abuse. Promoting awareness and embracing cultural diversity can create a safer and more compassionate society [7, 42].

Eleven items make up the third factor, which is care neglect. Intentional or unintentional unwillingness to provide physical or psychological care or failure to provide them with food, drink, or prescriptions are examples of neglect of care. Care neglect in the current study included: inattention, not making phone or personal interactions, failing to provide for and take care of necessities, and neglecting financial matters [43].

The other factor in this questionnaire addresses financial neglect. Financial neglect means neglecting to manage an older adult’s assets or failing to fulfill their financial obligations, such as paying their rent or mortgage, insurance premiums, energy bills, or property taxes [44, 45].

Ostracism was another factor that contributed to domestic elder abuse. In a recent study, older adults described rejection as annoying, painful, and inexplicable. Leaving older adults in hospitals is an essential form of domestic elder abuse [46]. It is estimated that between 15 and 30% of older adults are left in hospitals by their family members; It is worth mentioning that this statistic does not include the older adults who are left on the street or in their homes and are taken to hospitals or charitable institutions [11, 12]. The abandonment of older adults in care and treatment centers such as hospitals is one of the primary forms of misbehavior toward older adults. The family members leave their elderly in the hospital seasonally, especially during long weekends, Christmas days, Carnival, and school holidays, and do not return to discharge them, which leads to severe emotional and physical effects on older adults [47, 48].

Finally, one should note that the instrument introduced in this study has some limitations. There are various types of abuse such as “financial exploitation”, “ignoring the rights of the elderly”, “desecration”, and “sexual abuse”. Obviously, the SF-DEASQ instrument does not cover all of these forms. There are no items on “financial exploitation”, there are no items on “desecration”, “sexual abuse”, and there are hardly items on “ignoring the rights of the elderly” (although there may be some degree of overlap especially with “ostracism”).

## Conclusions

Given the trade-offs between the two, we believe this short form (27 items) of domestic elder abuse provides an excellent complement to the comprehensive (49 items) version of domestic elder abuse. We advise investigators to use the version best suitable for their project at each time point. The use of multiple forms of analysis to develop the short-form version of domestic elder abuse allows for more substantive validity of the results despite the limitations of each approach. The short form of domestic elder abuse is highly correlated with the comprehensive (49-item) version of domestic elder abuse and has high internal consistency. Overall, the SF-DEAQ represents a valuable contribution to the field of elder abuse research and has the potential to aid healthcare professionals, researchers, and policymakers in addressing the complex and pressing issue of domestic elder abuse.

## Data Availability

The datasets used and/or analyzed during the current study are available from the corresponding authors on reasonable request.

## List of abbreviations

CVI: Content Validity Index
CVR: Content Validity Ratio
EFA: Exploratory Factor Analysis
ICC: Intraclass Correlation Coefficients
KMO: Kaiser–Meyer–Olkin
MDC: Minimal Detectable Change
MIC: Minimal Important Change
SCVI/Ave: Scale Content Validity Index/ Average
SEM: Standard Error of Measurement
